# Seasonal and ontological variation in diet and age‐related differences in prey choice, by an insectivorous songbird

**DOI:** 10.1002/ece3.9180

**Published:** 2022-08-11

**Authors:** Sarah R. Davies, Ian P. Vaughan, Robert J. Thomas, Lorna E. Drake, Angela Marchbank, William O. C. Symondson

**Affiliations:** ^1^ Cardiff School of Biosciences Cardiff University Cardiff UK

**Keywords:** age comparison, dietary overlap, metabarcoding, niche space, ontological shift in diet, seasonal effects

## Abstract

The diet of an individual animal is subject to change over time, both in response to short‐term food fluctuations and over longer time scales as an individual ages and meets different challenges over its life cycle. A metabarcoding approach was used to elucidate the diet of different life stages of a migratory songbird, the Eurasian reed warbler (*Acrocephalus scirpaceus*) over the 2017 summer breeding season in Somerset, the United Kingdom. The feces of adult, juvenile, and nestling warblers were screened for invertebrate DNA, enabling the identification of prey species. Dietary analysis was coupled with monitoring of Diptera in the field using yellow sticky traps. Seasonal changes in warbler diet were subtle, whereas age class had a greater influence on overall diet composition. Age classes showed high dietary overlap, but significant dietary differences were mediated through the selection of prey; (i) from different taxonomic groups, (ii) with different habitat origins (aquatic vs. terrestrial), and (iii) of different average approximate sizes. Our results highlight the value of metabarcoding data for enhancing ecological studies of insectivores in dynamic environments.

## Graphical abstract

We compared diet and prey selectivity between three age classes of the reed warbler (*Acrocephalus scirpaceus*), an insectivorous, migratory songbird, at a breeding site in Somerset the United Kingdom. Our study was conducted over three stages of the summer breeding season to examine seasonal effects. It was discovered that birds of different ages showed considerable dietary overlap but partitioned prey according to habitat (terrestrial vs. aquatic), coarse prey size‐classes and taxonomy of prey, with different “preferences” for Dipteran families exhibited by different reed warbler age classes. We also found weaker evidence for seasonal changes in reed warbler diet.

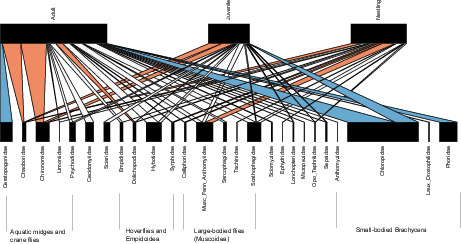

## 1 INTRODUCTION

Animals are under selective pressure to choose optimal food resources, to balance the time and energy costs of foraging (Bautista et al., 1998; Davies, 1977; Krebs, 1973; Pyke et al., 1977). In addition, as an individual matures over time, its nutritional requirements are likely to change to coincide with new challenges encountered.

Comparative studies focusing on the diet of different age classes of animals in the same location are lacking, although developmental (ontological) shifts in diet have been documented in vertebrates such as reptiles (Brown et al., 2014; East & Ligon, 2013; Elsey, 2006; Sloan et al., 1996), and seabirds (Alonso et al., 2014; Navarro et al., 2010). Dietary variation may not always be solely due to age, as diets may instead deviate during distinct life history events, such as offspring rearing. For example, in some bat species, lactating individuals consume different prey from non‐lactating individuals (Kunz et al., 1995; Lee & McCracken, 2002; Li et al., 2021). Together, the current literature suggests that age‐dependent or at least life‐stage‐dependent dietary change could be relatively common among vertebrates. Understanding and quantifying such ontological shifts may be essential for designing effective conservation measures that consider animals at all developmental stages.

If predators optimize their diets by selecting prey based on their nutritional content, then the diet of individuals of different age classes may be driven by selecting prey items that satisfy age‐specific nutritional requirements. To optimize reproductive success, adults must provision their offspring with prey that are sufficiently high in calories, protein, and a range of other nutrients for growth and development. This enables offspring to grow rapidly, leave the care of their parents earlier, and maximize their chances of survival (Krupa, 2004; Li et al., 2021; Orłowski et al., 2017). Prey with “breeding currency” are defined as prey of high‐value for breeding, that is, beneficial for offspring development, contributing to improved reproductive productivity (Greenberg, 1995; Yard et al., 2004). The concept of breeding currency originated from observations of adult birds feeding their broods with diets of caterpillars and other nutrient‐rich prey (e.g., Araneae, soft‐bodied Hemiptera, or the larvae of other insect orders) that are usually large in size (e.g., >5 mm in length), soft‐bodied, and/or easily digestible (Ceia et al., 2016; Johnson et al., 2005; Krupa, 2004; Orłowski et al., 2014; Xiong & Lu, 2014).

These same prey characteristics may not reflect the optimal foraging strategy of adult or juvenile (i.e., newly independent) animals. For insectivorous birds, the breeding productivity of adults is linked to the total abundance of breeding currency invertebrates that benefit the growth of their offspring (Greenberg, 1995; Johnson et al., 2005). However, the diet of adult and juvenile individuals themselves may be more directly related to the total abundance of all invertebrates available (Johnson et al., 2005) Adults may forage differently when searching for profitable prey for offspring than when feeding themselves (Jedlicka et al., 2017; Li et al., 2021). Selected prey for older age groups might offer specific nutritional benefits or better meet conditions for optimal foraging due to their abundance or nutritional composition (Greenberg, 1995; Johnson et al., 2005).

Foraging experience and learned foraging behavior can also influence diet. For example, young animals are inexperienced and may not yet have developed the required dexterity or prey‐handling abilities to capture large or highly mobile prey items (Marchetti & Price, 1989). Moreover, adults that are defending territories may exhibit dominance over juveniles, competitively excluding them from accessing higher‐quality foraging habitats (Wunderle, 1991).

The availability of invertebrates, as a food resource for insectivores, shows high spatio‐temporal variation and is dependent on temperature, particularly in seasonal climates (Aldridge & Rautenbach, 1987; Whitaker, 1994). If the overall abundance of high‐quality prey becomes limited during breeding, then reproductive success may be impaired (Rodenhouse & Holmes, 1992; Sillett et al., 2000; Trevelline et al., 2016). Individuals must thus adapt their foraging strategies to changes in the availability of different prey taxa.

Migratory insectivores such as songbirds are expected to show flexibility in their diet, as both the abundance and composition of the prey communities encountered at the multiple locations visited along the migration route are likely to vary. A foraging strategy of opportunistically consuming the most abundant or profitable prey in each time or location is thus likely to be beneficial for fitness.

**FIGURE 1:**
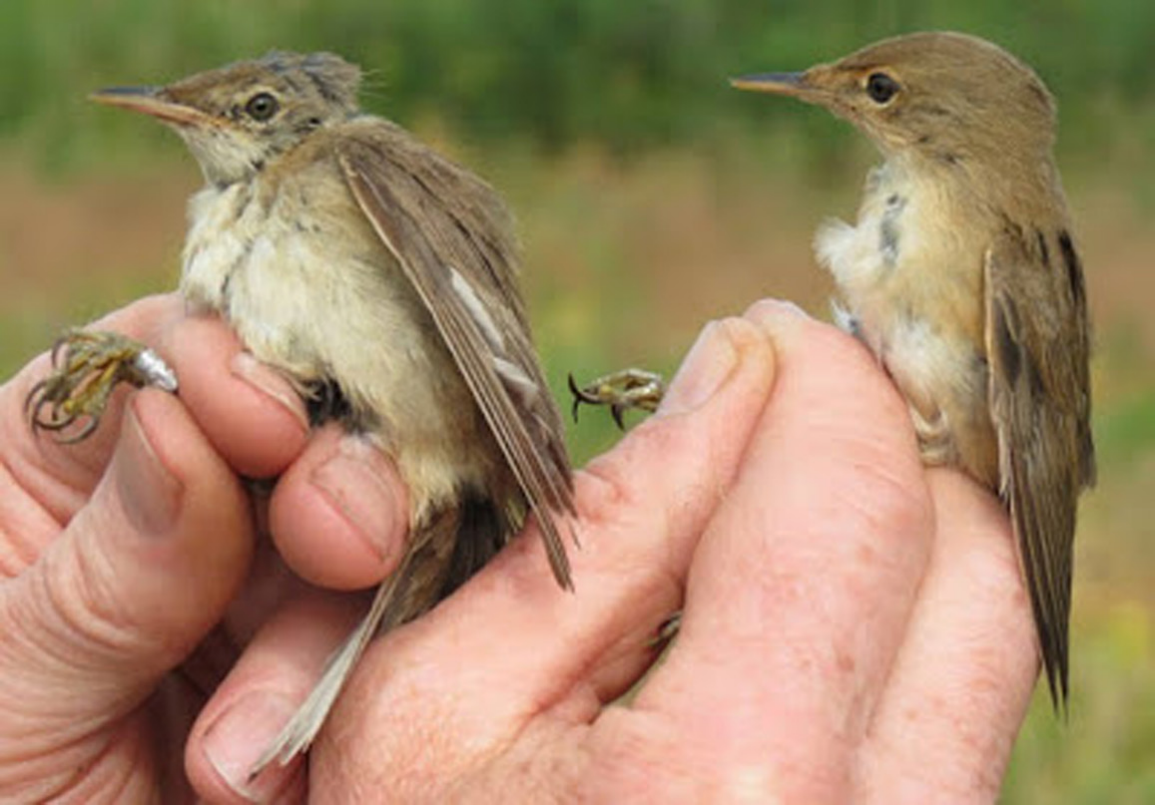
Cover photograph. A juvenile (left) and adult (right) reed warbler (*Acrocephalus scirpaceus*). Photographed by the Gower Ringing Group.

One migratory species that is thought to be able to modify its diet to track changes in prey resources is the Eurasian reed warbler (hereafter “reed warbler”), *Acrocephalus scirpaceus* (Figure 1). In the reedbeds of their north‐western European range, invertebrate emergence from wetted zones is seasonal, that is, reduced in the spring/early summer months compared to the middle/late summer months (Halupka et al., 2008; McKee & Richards, 1996; Vafidis et al., 2016). Common prey groups such as aquatic Diptera reach their peak during mid‐summer (Bibby & Thomas, 1985) but remain in high numbers until the end of summer, while mayflies and damselflies show sharper peaks in availability (Bibby & Green, 1983; Bibby & Thomas, 1985). The early summer is marked by an abundance of Lepidoptera larvae, Araneae, and Coleoptera, whereas late summer is characterized by aphids (Chernetsov & Manukyan, 2000).

Reed warblers are expected to show dietary flexibility, and studies suggest that they consume different prey as the composition and diversity of prey communities changes over the summer (Bibby & Thomas, 1985; Chernetsov & Manukyan, 2000; Vafidis et al., 2016). This species thus represents an excellent candidate species on which to track dietary composition and prey choice over time, potentially providing a model for other migratory passerines or insectivores with similar ecologies. Moreover, the reed warbler is currently experiencing changes to its life history as a result of climate change (Halupka et al., 2008; Kovács et al., 2012; Schaefer et al., 2006). Future alterations to prey communities as a result of warmer spring and summer temperatures could therefore have implications for the foraging behavior of the reed warbler as well as many other insectivorous species. Several publications have examined the diet of *Acrocephalus* species during various life stages to include the challenges of growth and development, dispersal and territory formation, reproduction, chick‐rearing, and migration (Bibby & Green, 1983; Chernetsov & Manukyan, 2000; Dyrcz & Flinks, 2000; Ezaki, 1992; Kerbiriou et al., 2011). Knowledge of such dietary requirements in this species (along with others), could help inform important conservation measures to improve prey abundance. Until recently however, it has been difficult to compare the diets of insectivorous species at different life stages at fine taxonomic detail, without expertise in morphological identification of prey remains in feces. The use of dietary metabarcoding has greatly improved the species‐level detection of prey in avian fecal samples (Jedlicka et al., 2017; Shutt et al., 2020; Trevelline et al., 2016, 2018). These new developments now allow diets to be compared across the age classes of a target study species.

We used high‐throughput sequencing (HTS) to compare the diets of nestlings, fledged juveniles, and adult reed warblers, at a wetland site in the United Kingdom over the 2017 summer breeding season. We also assessed the extent of dietary overlap between these age classes and measured prey availability of a major prey order—Diptera—in the field to compare the dietary preferences of the three age classes. While reed warbler diet has been characterized in the past by identification of hard parts in prey remains (Davies & Green, 1976; Grim, 2006; Grim & Honza, 1996) and more recently by qPCR sequencing (King et al., 2015), this is to our knowledge the first time that reed warbler diet has been successfully characterized by metabarcoding.

As our study is largely exploratory, we aim to address the following overarching question:

*How will diet composition differ (i) between age classes and (ii) over the breeding season?*

We predict that each age class will show significant differences in diet with regard to the orders, families, or species of prey consumed. This will be tied to the existence of specific preferences for different Dipteran taxa by adults, juveniles, and nestlings. We predict that the diet of all age classes will change over the course of the breeding season to mirror changes in local abundance and composition of prey taxa.

Furthermore, we hypothesize that nestlings will be fed larger prey species on average than adults or juveniles, and that Lepidoptera, as a breeding currency prey item, will be detected more frequently among nestling diet samples compared to adults or juveniles. We also hypothesize that dietary overlap will be high among all age classes but especially between adults and juveniles in the middle of the breeding season when prey availability is likely to be at its peak (allowing greater sharing of prey species). Since aquatic insects are often important components of songbird diet (Baxter et al., 2005; Stanton et al., 2017; Trevelline et al., 2016), we hypothesize that there will be no significant differences in the proportion of prey items that are classed as either aquatic or semi‐aquatic between age groups. Finally, we hypothesize that warblers will show measurable preferences for Dipteran prey that are large‐bodied and from aquatic habitats.

## 2 MATERIALS AND METHODS

### 2.1 Study site

**FIGURE 2:**
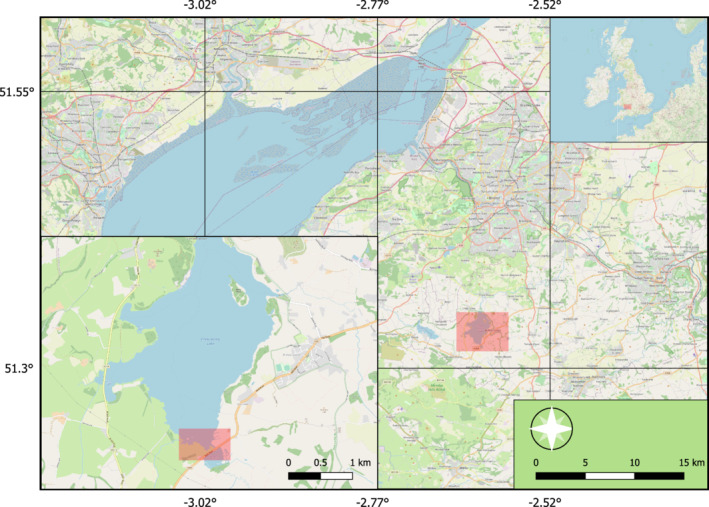
Location of Chew Valley Lake in Somerset, the United Kingdom (highlighted in red and expanded in the bottom left corner). The study area, incorporating both CES locations, is also highlighted.

Chew Valley Lake (Grid Ref: ST5659) is a large (approx. 486 ha) man‐made lake located at the northern edge of the Mendip Hills in Somerset, the United Kingdom (Figure 2). The lake is surrounded by grassland, scrub, and wet willow (*Salix* spp.) “carr” woodland (i.e., woody species, low growing plant species, and shade‐tolerant herb species) with extensive *Phragmites australis* reedbed habitat fringing the water's edge. The site also comprises hedge‐bounded pasture grazed by low densities of cattle. The lake is designated as a Site of Special Scientific Interest (SSSI) and a Special Protection Area (SPA). The Chew Valley bird ringing station has been active on‐site since the 1960s and ringing activities take place year‐round.

### 2.2 Sample collection

Collection of reed warbler fecal samples took place during weekly bird ringing sessions at Chew Valley Lake (April–August 2017), as part of the British Trust for Ornithology's (BTO) constant effort sites scheme (CES). Visits were split between two ringing locations situated on opposite sides of the southern portion of the lake (Figure 2). Mist nets were erected through sections of reedbed and surrounding scrub, and were open from 30 min before dawn, until midday. Captured birds were aged as juvenile (birds hatched in the current year) or adult (birds hatched at least one calendar year before) based on plumage and molt patterns. We checked that the wings of any juveniles were fully grown to avoid collecting samples from fledged birds that may still be fed by parents. Adult and juvenile birds were placed into individual clean, breathable cotton bags for up to 15 min or until defecation (to minimize stress) after which they were ringed and released. To prevent cross‐contamination, bags were not used more than once, and a new pair of sterile forceps was used to collect fecal samples from each bag. Samples were placed in 1.5‐ml Eppendorf tubes with 100% molecular grade ethanol.

Between May and early August, a bird‐ringer with a license endorsement to ring pulli (nestlings) located reed warbler nests (*n* = 40) via systematic searching and collected fecal samples from nestlings. Nestlings were gently removed from the nest to be ringed, and any fecal sacs produced were collected directly and immediately transferred to 100% ethanol in 2‐ml Eppendorf tubes. Only nestlings of a ringable age (between 7 and 10 days old) were handled, to prevent premature fledging. Samples were only collected from an individual nest once over the summer, at the same time they were ringed. This avoids repeated disturbance to nests and minimizes the risk of predators locating nests. All fecal samples were stored in a laboratory freezer at −80°C at Cardiff University.

### 2.3 Invertebrate availability

Double‐sided (dry‐stick) 10 × 25 cm yellow invertebrate traps, henceforth “sticky traps,” were used to monitor Diptera abundance at both ringing locations (Oecos UK; www.oecos.co.uk/sticky‐traps‐drystick/). These traps measure the activity‐density of (mainly) flying insects and are particularly effective for Diptera (Black & Krafsur, 1985; Goulson et al., 2005; Hogsette et al., 1993) a group representing a large portion of known reed warbler diet (Davies & Green, 1976; Grim, 2006; Grim & Honza, 1996; King et al., 2015). Traps were enclosed in a plastic wire gardening mesh with holes 1 × 1 cm to prevent consumption or damage of trapped specimens by birds, and to prevent injury to small birds and mammals.

Sticky traps were deployed three times over summer to capture early (late May), middle (late June), and late summer (early August) abundances of Diptera. They were in close vicinity to the mist nets; five in reedbed and five in scrub habitats and attached to vegetation at heights between 0.5 and 2 m to cover the variation in reed warbler feeding heights. Traps were collected 7 days after deployment in the absence of rain, or up to 10 days if significant rainfall (more than three consecutive days of rainfall >1 mm) occurred, to correct for weather‐related biases in capture effectiveness. Individuals from 25% of both sides of the full‐size trap were identified to family level with the aid of a microscope and taxonomic keys. Due to the difficulty of achieving accurate identification to family for some Dipteran groups, several families were merged (Opo_Tephritidae = Opomyzidae/Tephritidae, Laux_Drosophilidae = Lauxanidae/Drosophilidae, Music_Fann_Anthomyiidae = Musicadae/Fanniidae/Anthomyiidae, see Appendix S1: S6 for details).

### 2.4 Molecular lab work

Samples from this study were processed and analyzed as part of a larger study on warbler diet (Davies, 2020). Details of steps taken to avoid contamination during laboratory work are included in Appendix S1: S1.

DNA extractions from the fecal samples were performed in batches of 23 using the QIAamp® DNA Stool Mini Kit from Qiagen. Each extraction round contained two extraction negatives to test for contamination among samples. The kit protocol with modifications from Zeale et al. (2011) were followed with additional customization steps (described in Shutt et al., 2020; see Appendix S1: S1 for details). The modifications adjusted for the smaller size of warbler feces (<200 mg) and higher levels of PCR‐inhibiting uric acid present. These were found to improve DNA yields when compared to the standard protocol.

Where possible, removal of surrounding uric acid from samples was undertaken before DNA extraction. Since nestling fecal samples were collected as a whole fecal sac, they were encased in a higher amount of uric acid than adult and juvenile samples. Removal of uric acid improved amplification success of prey DNA, validated by comparisons of PCR success (visualized by gel electrophoresis) from rounds where uric acid was not removed, and rounds where uric acid was entirely removed.

We used the general COI primers, mlCOlintf, 5′ACGCTCGACAGGWACWGGWTGAACWGTWTAYCCYCC3′ (Leray et al., 2013) and Nancy, 5′ACTAGCAGTACCCGGTAAAATTAAAATATAAACTTC3′ (Simon et al., 1994), which amplify a ~384 bp region of DNA. This combination of both Stockdale (2018) first used the forward and reverse primer to examine thrush diet, after extensive testing in silico and in vitro. This pair successfully isolates invertebrate DNA, enabling identification to species level, but does not amplify the DNA of the avian host (Stockdale, 2018). We tested these primers on 18 invertebrate orders and 50 families, and ~92% of tested taxa were successfully amplified (Davies, 2020). In addition, the majority of known invertebrate species from mock community mixes were successfully recovered after HTS sequencing (Appendix S1: S3).

Presence of target prey DNA in the fecal sample extracts was confirmed with PCR, using a Multiplex PCR Kit (Qiagen). Results were viewed under ultraviolet (UV) light via a transilluminator, on a 2% agarose gel. All DNA samples that amplified successfully in the initial PCR step were screened again in PCRs with forward and reverse primers labeled with MID‐tags (Multiplex Identifiers in the form of unique DNA tags), comprising 10 unique base pairs created using a custom oligos design process (Eurofins Genomics). Twenty‐four unique forwards and fifteen unique reverses (Appendix S1: S2) were used in combination so that each sample could be differentiated after pooling (Brown et al., 2013).

In each reaction well of each 96 well PCR plate, 2.5 μl of forward and reverse MID‐tag primer (2 μM), 12.5 μl of Qiagen Multiplex PCR Master Mix, 2.25 μl of nuclease‐free water and 0.25 μl of BSA were combined in a DNA‐free zone under an air flow hood. Five microliters of DNA from each sample were added to each well to give a total reaction volume of 25 μl. Per plate, we included with the DNA samples (i) extraction negatives (assigned at random across the plate), (ii) a column of PCR negatives (nuclease free water) to test for the presence of contamination in each row, (iii) at least one positive control (DNA extracts from the tissue of a shrimp [family: Penaeidae] and a mussel [family: Mytilidae]) and (iv) a mock community sample consisting of a mix of DNA from known reference invertebrates added at different concentrations (Appendix S1: S3). The PCR reaction took place for approximately 3 h on a SimpliAmp thermocycler and included a hot start at 95°C for 15 min, proceeding with 35 cycles of 94°C for 30 s, annealing at 55°C for 90 s, extension at 72°C for 90 s followed by 72°C for 10 min.

Samples were pooled by plate according to concentrations (ng/μl) given by Qiaxcel Advanced System (Qiagen) and equalized to roughly equimolar concentrations. Pools were cleaned using SPRIselect beads (Beckman Coulter), removing products <350 bp in length in a ratio of 0.9 (to retain fragments between ~350 and 1000 bp) and pooled DNA concentration was quantified using Qubit dsDNA High‐sensitivity Assay Kits. Quality checking was completed via TapeStation 2200 (Agilent) and each PCR plate pool was pooled to create one equimolar indexing “super pool.” Sequence libraries were prepared for Illumina MiSeq using the NEXTflex™ Rapid DNA‐Seq Kit (1 ng–1 μg) V15.10 (Bioo Scientific), compatible with the Illumina sequencing platform. The final pooled and indexed product was sequenced using a v3 MiSeq Reagent kit.

### 2.5 Bioinformatics and data processing

The bioinformatics pipeline followed Drake (2020) and Drake et al. (2021) (scripts and pipeline steps in Appendix S1: S4). Trimming, aligning, and quality checks were performed using FastP v.0.20.0 (Chen et al., 2018), tagged reads were labeled by sample id using Mothur (Schloss et al., 2009) and demultiplexed to one file per sample. The Unoise3 command (Edgar, 2016; Edgar & Flyvbjerg, 2015) in Usearch v.11 (Edgar, 2010) was selected to (i) remove chimeras and noise, (ii) cluster the reads to generate denoised zero‐radius OTUs (zOTUs) using a clustering threshold of 100%, and (iii) create a read abundance matrix for samples and zOTUs. The Blastn algorithm (Altschul et al., 1990) in Blast+ (Camacho et al., 2009) assigned taxonomic identities to the zOTUs, by comparing focal sequences to reference sequences held in NCBI GenBank. We identified unique dietary items using the top hit for each zOTU based on bit‐score, using MEGAN6 (Huson et al., 2016). A minimum percentage identity score of 97% was required to assign a species‐level match, otherwise zOTUs were classified to genus or family.

Our clean‐up process served to minimize artifacts from tag‐jumping and contamination. Data clean‐up was carried out with a suite of data processing methods, following guidance by Drake et al. (2021) and Cuff et al. (2021) (Appendix S1: S4). Rather than using a set of read threshold for retaining a zOTU in a sample (e.g., the 10 reads commonly used by studies), we determined thresholds for read removal using the percentage at which known artifacts assigned to the known mock community samples were removed, while still retaining known species added to the mix (Cuff et al., 2021; Drake et al., 2021). For our diet matrix, the read count of a zOTU needed to exceed a threshold of 0.2% of the *maximum read count for all sequences from that sample* and more than 0.5% of the *maximum read count for all incidences of that zOTU across all samples*, to be retained. We performed this step to reduce the incidence of tag‐jumping from zOTUs with very high read counts across multiple samples. In addition, we manually removed several known lab contaminants and nondietary species (listed in Appendix S1: S5).

Dietary data from one individual nestling per nest was selected at random for inclusion in the study to avoid potential pseudo‐replication in the next steps. Frequency of occurrence (FOO) of a prey taxon was calculated by summing the number of instances that a given taxon occurred across all sampled reed warblers within age and/or season groups. This was then expressed as a percentage of the total number of individuals (% FOO). Data were organized by “season”: either “early” (23rd April–30th May), “middle” (1st June–29th July), or “late” (6th August–19th August), which corresponded with the date the individual bird was captured and sampled. These three periods matched the timescale of the three sampling periods during which the invertebrate monitoring occurred.

### 2.6 Data analysis

All analyses were carried out using R version 4.05 (R Core Team, 2018), implemented in RStudio version 1.4.1106 (RStudio Team, 2021). General linear models (GLMs) were checked for fit and model assumptions validated with appropriate tests; we kept our approach consistent between models. Unless otherwise stated, the variables of all GLMs were checked for significance using *ANOVA* tests and the drop1 function based on model AIC. When carrying out large numbers of tests between factor levels (e.g., ages subset by season), pairwise differences between the dependent variables were measured with posthoc Tukey tests using the package *emmeans* (Lenth, 2020), which adjusts the *p*‐value for multiple comparisons. Since adults, juveniles, and nestlings did not co‐occur in all seasons, we included the interaction between season and age in all models and only test for differences between samples of different age groups during the same season (e.g., adults and nestlings in early summer are tested separately from mid‐summer).

#### 2.6.1 Diet composition

Multivariate analysis of dietary composition was carried out using the package *mvabund* (Wang et al., 2012). We fitted the presence/absence data of all prey taxa at (i) the family level and (ii) order level (suborder for Diptera) to a binomial family *manyglm* model with a complementary log–log (“cloglog”) link function. *Manyglm* fits a single GLM to each response variable with a common set of predictor variables, in our case; age, season, and the interaction between them. Predictor variables were tested with the *ANOVA* function which resamples the data to test for significant community level responses to the predictors using likelihood ratio tests. The univariate test option within *ANOVA* was applied to identify any significant relationships between the test variables and specific prey taxa within the diet matrix. Nonmetric multidimensional scaling (NMDS) based on Jaccard dissimilarities was used to visualize the differences in diet among ages and seasons using the package *vegan* (Oksanen et al., 2018).

For each dietary sample, prey items were divided into two classes; (i) “aquatic” here encompassing both semi‐aquatic and aquatic prey groups, classified as having one or more aquatic stage in the life cycle, and (ii) “terrestrial” encompassing all nonaquatic prey taxa (i.e., the remaining prey after removal of aquatic species). Variation in the proportion of aquatic invertebrate species detected in reed warbler diets was examined across ages, season, and their interaction using a binomial GLM with a logit link function, weighted by the number of unique prey items. We checked for overdispersion by calculating theta, defined as the model residual deviance/residual degrees of freedom.

Since it is not possible to directly measure the size of each species of prey present in the fecal samples, we systematically searched entomological literature, specialist websites, and invertebrate keys to find approximate body lengths in millimeters for each dietary species detected (Appendix S1: S7). For consistency, the body lengths for adult stages (rather than nymphs or different instars of larvae) were used, as life stage information about dietary items was unknown. However, before proceeding we checked that the body lengths of larvae were not greatly different from adult body lengths. The average prey size of all items in each fecal sample for each bird was then calculated. Differences in average prey size between adults, juveniles, and nestlings were determined using a Gaussian GLM with an “identity” link function with the additional predictor variables, season, and the interaction between age and season.

#### 2.6.2 Dietary overlap and prey choice models

To quantify overlap in observed reed warbler diets, we used the *EcoSimR* package (Gotelli et al., 2015) to calculate Pianka's measure of dietary overlap (Pianka, 1973) between pairs of age classes. Pianka's overlap quantifies the degree of similarity between two diets with complete overlap assigned a value of 1 and no overlap and complete dietary partitioning assigned a value of 0. In *EcoSimR*, a null model simulation (command = niche_null_model), based on randomization of dietary data (here based on FOO) is generated and used in a statistical comparison with the “observed model” (our inputted diet data as FOO), with significant deviations from the null model indicating either significant dietary overlap (positive standardized effect sizes [SES]) or significant partitioning (negative SES). We used randomization algorithm 3 (RA3) which is a permutation test recommended for niche overlap studies that randomly switches the number of consumed prey among prey species for each bird group. Pairwise analyses were performed for adults, juveniles and nestlings with the simulation set to run for 9999 repetitions. We also ran separate simulations subsetted for birds captured during early, middle, and late summer to determine any effect of season. Our observed data in each case formed a matrix (prey species in columns and bird groups in rows) to include the number of birds of each age group or age: season group in which each prey species was found.

We characterized the dietary preferences of each warbler age class using null modeling with the R package *econullnetr*, which was developed to identify prey choices by predators (Vaughan et al., 2018). It uses a null‐modeling approach to compare the observed frequency of occurrence of prey items in the diet to the frequency expected based upon their recorded abundance in the field. The null model simulates the diet in the absence of prey choice, whereby the frequency with which a prey taxon is consumed is simply a consequence of how abundant that taxon is in the field. We restricted the analysis to Dipteran taxa, since sticky traps sample Diptera effectively, and we are confident that our measure of abundance closely reflects the true availability of this order to warblers. We did not include other prey orders in this analysis because yellow sticky traps show taxonomic biases, for example, underestimating Lepidoptera and Gastropoda (Thomson et al., 2004). Dipteran families present in the diet of one or more warbler age group, but not recorded in the field, were removed from the analysis.

Within *econullnetr*, a null model simulation (generate_null_net) was created based on (i) our diet data for each individual reed warblers, representing which Diptera prey families each individual had consumed, subset by age and season, and (ii) prey abundance data, that is, number of individuals of each Diptera prey family identified on all sticky traps combined. The model was run for 9999 iterations. Taxa that occur in the diet significantly more frequently than expected from the null model simulation (i.e., that expected from their abundance alone) indicate preferred food items, while those occurring less frequently than expected indicate undesirable prey items (Vaughan et al., 2018).

## 3 RESULTS

One hundred and forty reed warbler fecal samples were collected, including 65 nestling samples collected from 42 nests. In total, 102 samples produced dietary data, giving an overall success rate of 71.4% although the success rate for nestlings (64%) was lower than that of adults and juveniles (80%). After randomly selecting one nestling sample per nest, the total number of samples was 90, comprising 31 nestlings (4 in early summer and 27 in mid‐summer), 20 juveniles (14 in mid‐summer and 6 in late summer), and 39 adults (14 in early summer, 21 in mid‐summer and 4 in late summer).

One hundred and ninety‐two prey species from 94 invertebrate families and 11 orders were identified in the diet (Appendix S4). Seventy‐two species were detected from nestling samples, 56 from juveniles, and 95 from adults. Fecal samples yielded a mean of 7.5 unique prey taxa ±3.9 SD (range = 3–23). Diptera was the most frequently detected order, present in 85.5% of samples (Table 1). The remaining orders with the highest % FOO were; Lepidoptera (30%), Hemiptera (22.2%), Araneae (20%), Hymenoptera (20%), and Coleoptera (18.9%). Nestling diet consisted of fewer prey species overall, including larger‐bodied groups such as gastropods, Odonata, and Lepidoptera at more elevated frequency. Juveniles consumed fewer species of spiders (Araneae), and these were detected in fewer juvenile samples compared to the other age classes. Adults consumed the highest diversity and % FOO of Diptera, Coleoptera, Hemiptera, and Araneae.

**TABLE 1:** Percentage frequency of occurrence (% FOO) and species richness (number of unique prey taxa—shown in brackets) of invertebrate orders detected in adult, juvenile, and nestling reed warbler diet samples and all samples combined. Values are color coded according to frequency of occurrence (darker shade of color = higher abundance).

Order	% Frequency of occurrence (species richness)			
	Adult (*n* = 39)	Juvenile (*n* = 20)	Nestling (*n* = 31)	All (*n* = 90)
Acari	0	5 (1)	0	1.11 (1)
Araneae	25.64 (5)	10 (1)	19.35 (7)	20 (10)
Coleoptera	28.21 (10)	20 (5)	6.45 (2)	18.89 (14)
Diptera	92.31 (62)	85 (33)	77.42 (34)	85.56 (96)
Ephemeroptera	0	5 (1)	3.23 (1)	2.22 (1)
Gastropoda	5.13 (3)	5 (2)	16.13 (4)	8.89 (6)
Hemiptera	33.33 (11)	10 (2)	16.13 (5)	22.22 (15)
Hymenoptera	33.33 (18)	20 (5)	3.23 (1)	20 (22)
Lepidoptera	17.95 (7)	15 (3)	54.84 (14)	30 (20)
Neuroptera	2.56 (1)	5 (1)	6.45 (2)	4.44 (3)
Odonata	5.13 (1)	0	12.90 (1)	6.67 (1)
Trichoptera	5.13 (1)	40 (2)	6.45 (1)	13.33 (2)

The prey species detected in the diets of reed warblers varied according to age, but the most frequently detected species, found in all age classes, was the phantom midge *Chaoborus flavicans*. This species was present in 46.7% of samples, followed by the chironomids; *Chironomus* sp. in 25.6% of samples and *Endochironomus albipennis* in 15.6% samples, a dungfly *Scathophaga stercoraria* in 15.6% of samples and a caddisfly *Oecetis ochracea* in 13.3% of samples (Table 2).

**TABLE 2:** Percentage frequency of occurrence (% FOO) of the top 40 most common invertebrate species detected in adult, juvenile, and nestling reed warbler diet samples. Values are color coded according to frequency of occurrence (darker shade of color = higher abundance).

Order	Family	Taxon	% Frequency of occurrence			
			Adult	Juvenile	Nestling	All
Diptera	Chaoboridae	*Chaoborus flavicans*	46.15	65	35.48	46.67
Diptera	Chironomidae	*Chironomus* sp.	35.90	25	12.90	25.56
Diptera	Chironomidae	*Endochironomus albipennis*	10.26	40	6.45	15.56
Diptera	Scathophagidae	*Scathophaga stercoraria*	17.95	5	19.35	15.56
Trichoptera	Leptoceridae	*Oecetis ochracea*	5.13	40	6.45	13.33
Diptera	Muscidae	*Helina* sp.	5.13	5	16.13	8.89
Diptera	Chironomidae	*Procladius sagittalis*	15.39	5	0	7.78
Araneae	Linyphiidae	*Hypomma bituberculatum*	7.69	10	3.23	6.67
Diptera	Chironomidae	*Cryptochironomus psittacinus*	10.26	5	3.23	6.67
Diptera	Hybotidae	*Platypalpus* sp.	12.82	5	0	6.67
Lepidoptera	Noctuidae	*Lenisa geminipuncta*	10.26	0	6.45	6.67
Odonata	Coenagrionidae	*Enallagma cyathigerum*	5.13	0	12.90	6.67
Diptera	Chironomidae	*Cladotanytarsus atridorsum*	10.26	5	0	5.56
Diptera	Chironomidae	*Prodiamesa olivacea*	12.82	0	0	5.56
Diptera	Dolichopodidae	*Dolichopus plumipes*	7.69	0	6.45	5.56
Diptera	Empididae	*Empis stercorea*	10.26	0	3.23	5.56
Diptera	Muscidae	*Helina depuncta*	5.13	0	9.68	5.56
Araneae	Clubionidae	*Clubiona phragmitis*	7.69	0	3.23	4.44
Coleoptera	Chrysomelidae	*Galerucella lineola*	5.13	5	3.23	4.44
Diptera	Chironomidae	*Chironomus pallidivittatus*	7.69	5	0	4.44
Diptera	Chironomidae	*Cricotopus sylvestris*	10.26	0	0	4.44
Diptera	Dolichopodidae	*Chrysotus femoratus*	7.69	5	0	4.44
Gastropoda	Succineidae	*Succineidae* sp.	2.56	0	9.68	4.44
Hemiptera	Aphididae	*Microlophium carnosum*	10.26	0	0	4.44
Hemiptera	Notonectidae	*Notonecta glauca*	2.56	0	9.68	4.44
Lepidoptera	Noctuidae	*Mythimna straminea*	2.56	0	9.68	4.44
Araneae	Linyphiidae	*Porrhomma pygmaeum*	7.69	0	0	3.33
Diptera	Anthomyiidae	*Delia florilega*	7.69	0	0	3.33
Diptera	Chironomidae	*Chironomus nuditarsis*	0	10	3.23	3.33
Diptera	Chironomidae	*Cryptochironomus obreptans*	7.69	0	0	3.33
Diptera	Dolichopodidae	*Dolichopus cilifemoratus*	0	0	9.68	3.33
Diptera	Muscidae	*Musca autumnalis*	0	5	6.45	3.33
Diptera	Opomyzidae	*Opomyza germinationis*	2.56	0	6.45	3.33
Diptera	Scathophagidae	*Scathophagidae* sp.	7.69	0	0	3.33
Gastropoda	Lymnaeidae	*Stagnicola* sp.	2.56	5	3.23	3.33
Hemiptera	Aphididae	*Pterocomma* sp.	7.69	0	0	3.33
Hemiptera	Cicadellidae	*Empoasca luda*	2.56	5	3.23	3.33
Lepidoptera	Noctuidae	*Orthosia cerasi*	0	0	9.68	3.33

Pianka's overlap index was greatest between adults and juveniles (0.65), followed by adults and nestlings (0.63), and lowest between juveniles and nestlings (0.59). All pairwise combinations showed significantly greater dietary overlap than predicted by the null model (*p* < .05, Table 3i).

**TABLE 3:** Pianka's index of niche overlap (*O*_*jk*_) in observed diet between reed warblers of the three age classes, (i) over the whole breeding season study period and (ii) during different stages or “seasons” of the breeding season. Cells are color coded according to whether the Pianka index indicated overlap was significant (green) or not significant (yellow). Standard effect sizes (SES) and *p*‐values are also indicated for each pairwise test with asterisks indicating the significance level of the test with respect to the null model.

(i)						
Age	Adult			Juvenile		
	*O*_*jk*_	SES	*p*‐Value	*O*_*jk*_	SES	*p*‐Value
Nestling	0.63	6.51	.0002***	0.59	4.90	.004**
Adult				0.65	7.28	.0003***

(ii)																		
	Adult									Juvenile								
	Early			Middle			Late			Early			Middle			Late		
	*O*_*jk*_	SES	*p*‐Value	*O*_*jk*_	SES	*p*‐Value	*O*_*jk*_	SES	*p*‐Value	*O*_*jk*_	SES	*p*‐Value	*O*_*jk*_	SES	*p*‐Value	*O*_*jk*_	SES	*p*‐Value
Nestling	0.26	−0.87	.24	0.64	5.20	.003**							0.55	3.91	.009**			
Adult													0.64	5.13	.003**	0.49	0.32	.43

*Note*: Asterisks denote significant deviations from the null model (*p < .05, **p < .01, ***p < .001). Green shade indicates higher observed overlap than the null model. Yellow shade indicates observed overlap is not significantly different from the null model.

In the early part of the breeding season, dietary overlap between adults and nestlings was much lower compared to the middle of the breeding season (Table 3ii), but it did not significantly differ from the null model (Pianka index = 0.26, *p* = .24). Similarly, by the end of the breeding season, dietary overlap between adults and juveniles was not significantly different to that predicted by the null model (Pianka index = 0.49, *p* = .43). Nestlings and adults showed a significant increase in dietary overlap from early to mid‐breeding season (Pianka index = 0.64, *p* = .003), comparable to the extent of overlap between adults and juveniles in the middle of the breeding season (Pianka index = 0.64, *p* = .003). Juveniles and nestlings only coexisted in the middle of the breeding season, when they showed significant dietary overlap (0.55, *p* = .009), but it was lower than the overlap between either adults and juveniles, or adults and nestlings.

In the *mvabund* higher taxon level analysis, only age was significant in predicting the diet composition of birds (LRT = 82.8, residual df = 86, *p* = .001, test statistics available in Appendix S2), significantly influencing dietary occurrences of nematocerous Diptera (LRT = 14.8, *p* = .008), Hymenoptera (LRT = 11.9, *p* = .02), Lepidoptera (LRT = 13.3, *p* = .01), and Trichoptera (LRT = 13, *p* = .02, Appendix S2). Nestling diet had a much higher incidence of Lepidoptera (55% of samples vs. 18% in adults and 15% in juveniles) but lower incidence of nematocerous flies (45% of samples vs. 84% of adults and 85% juveniles) and Hymenoptera (3% vs. 33% in adults and 20% in juveniles), whereas juvenile diet was associated with higher frequency of occurrence of Trichoptera (40% of samples vs. 6% in nestlings and 5% in adults).

Age (LRT = 250.3, residual df = 86, *p* = .001), season (LRT = 152.1, residual df = 84, *p* = .02), and the interaction between age and season (LRT = 52.3, residual df = 82, *p* = .006) were all significant predictors of reed warbler diet composition in the family level analysis (Appendix S2). Neither season nor the interaction between season and age affected the frequency of occurrence of any individual prey families, suggesting that any changes in diet composition due to seasonality involve subtle changes at the community level. The occurrences of the prey families Chironomidae (LRT = 17.07, *p* = .006) and Leptoceridae (LRT = 12.97, *p* = .03) were significantly influenced by age class, with Chironomidae featuring less prominently in nestling diet compared to that of adults and juveniles (FOO = 19.4% in nestlings but 64.1% and 60% in adults and juveniles), and Leptoceridae was consumed much more frequently by juveniles than by adults or nestlings (% FOO = 40% in juveniles and vs. 5.1% and 6.5% of adults and nestlings, respectively).

**FIGURE 3:**
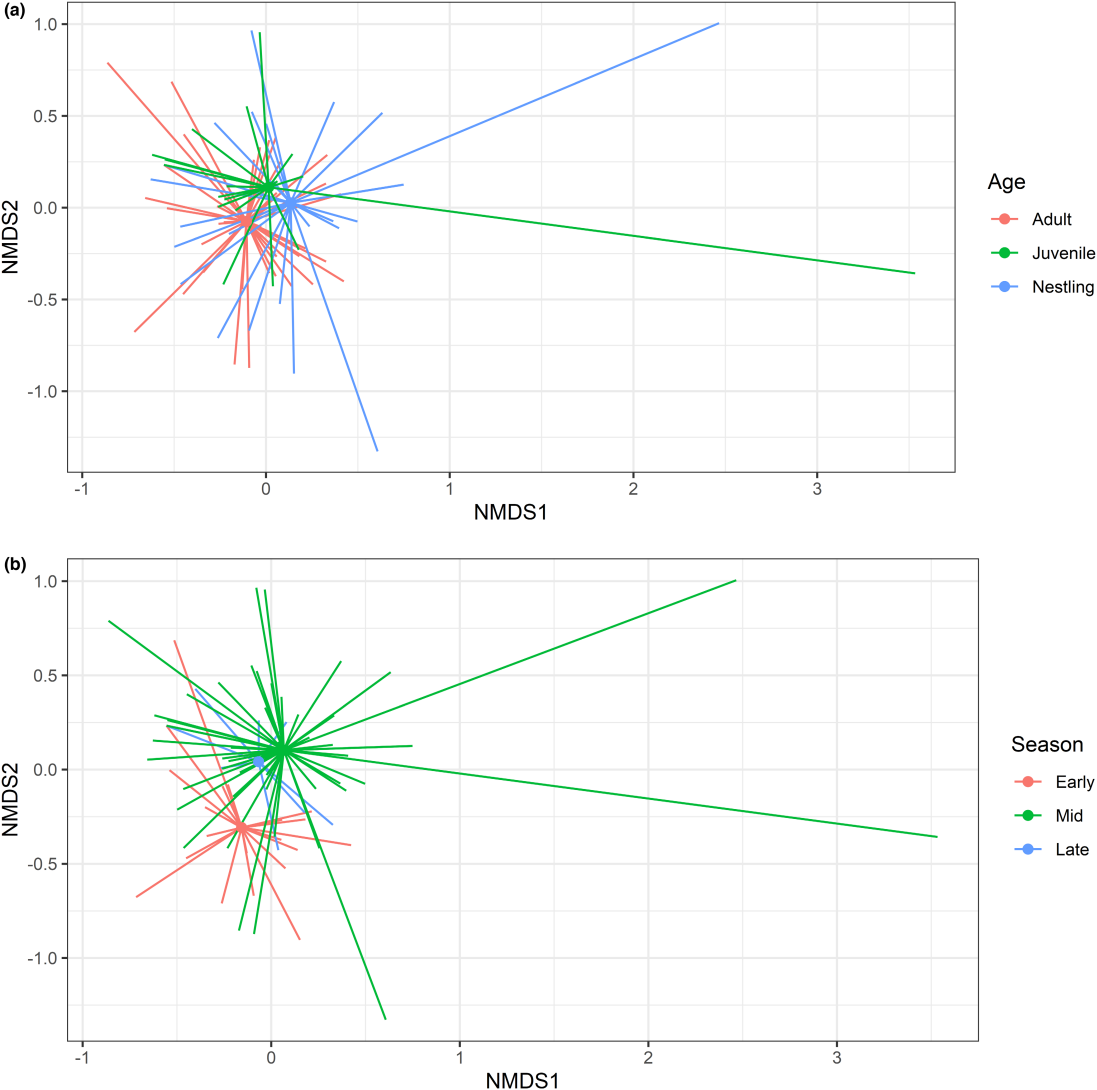
Nonmetric multidimensional scaling (NMDS) plot of the prey families detected in reed warbler diet samples collected at Chew Valley according to the significant parameters from the *manyglm* model: (a) age class, (b) season (early summer, mid‐summer, late summer). Stress = 0.15.

The NMDS plot based on family level indicated a degree of separation in consumption of prey families among age classes and seasons albeit with considerable overlap (Figure 3, stress = 0.15, *k* = 2 [prey community scores available in Appendix S1: S8]). In particular, birds captured in the early and late season showed a greater difference in dietary composition than birds captured in early and middle or middle and late season. In addition, nestling diet appeared more distinct than either adult or juvenile diet.

**FIGURE 4:**
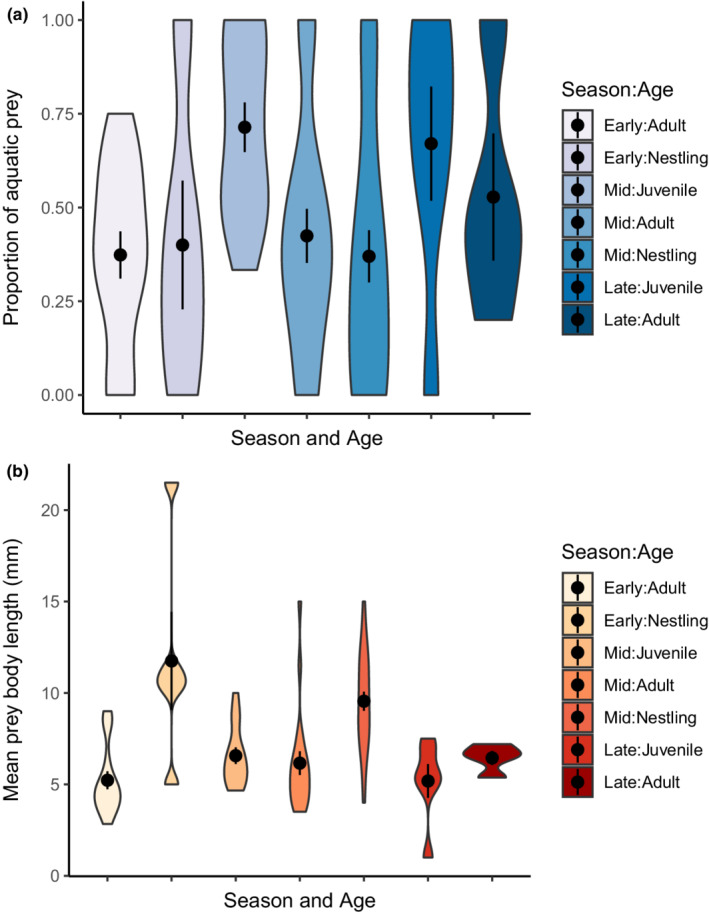
Violin plots showing (a) the proportion of prey items detected in the diet of Chew Valley reed warblers classed as aquatic or semi‐aquatic and (b) the average body length of prey (mm) detected in the diet samples, for adult, juvenile and nestling age classes subset by season; early = early summer, mid = mid‐summer and late = late summer. Means and standard errors for each factor level are displayed within the plot.

Reed warbler age (LRT = 124.1, df = 85, *p* = 1.92e‐05) was a significant predictor of the proportion of aquatic prey consumed by an individual in our binomial GLM (adjusted *R* ^2^ = .15, *F*_6,81_ = 3.49, *p* = .004**, *θ* = 1.5, Figure 4a), whereas season (LRT = 123.23, df = 83, *p* = .65) and the interaction between age and season (LRT = 121.76, df = 81, *p* = .48) was not significant. Adults and nestlings consumed a similar proportion of aquatic dietary items (early summer; *p* = .25, mid‐summer; *p* = .99), whereas juveniles consumed aquatic prey more frequently on average compared to both adults and nestlings in the mid‐summer (adults; *z* = 3.4, *p* = .02, nestlings; *z* = 3.8, *p* = .004) but not in the late summer (adults; *p* = .98, Figure 4). Age (*F*_2_ = 22.6, df = 613.6, *p* = 1796e‐08) also had a significant influence on the average size of prey consumed by reed warblers in our Gaussian GLM (adjusted *R* ^2^ = .33, *F*_6,81_ = 8.34, *p* = 3.732e‐07, Figure 4b), while season (*F*_2_ = 0.1, df = 612.28, *p* = .91) and the interaction between age and season (*F*_2_ = 2.5, df = 576.3, *p* = .09) were not significant. Nestling diets were formed of larger prey items on average compared to either adult (early summer; *z* = 4.7, *p* = .0003, and mid‐summer; *z* = 4.2, *p* = .002) or juvenile (mid‐summer; *z* = 3.3, *p* = .03) diets, whereas adult and juvenile diets did not differ in the average sizes of prey species consumed (mid‐summer; *p* = 1, late summer; *p* = .99).

**FIGURE 5:**
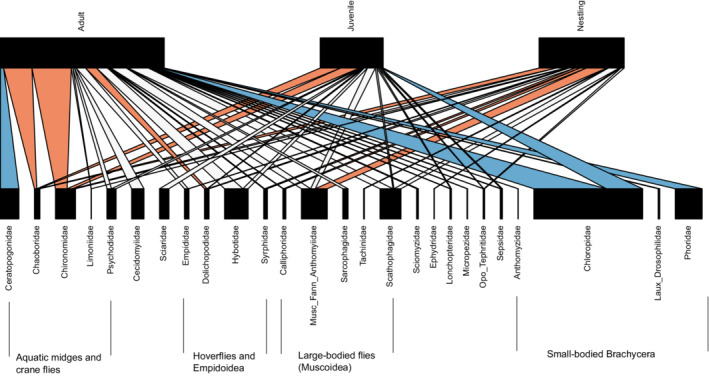
Bipartite food web plot illustrating the trophic interactions, and their strength, between warbler age classes and Diptera prey families from the *econullnetr* analysis. White, orange, and blue links indicate preferences for prey by warblers that were equal to, stronger or weaker, respectively, than that expected given the measured availability (abundance) of prey measured in the field. The width of the lower boxes is proportional to the availability of a particular prey family in the field, whereas the width of the top black boxes indicates the sample size for each warbler age group. Prey families are grouped according to taxonomy, with notable groups indicated. Key to merged families; Laux_Drosophilidae, = Lauxanidae/Drosophilidae, Music_Fann_Anthomyiidae = Musicadae/Fanniidae/Anthomyiidae, Opo_Tephritidae = Opomyzidae/Tephritidae.

**FIGURE 6:**
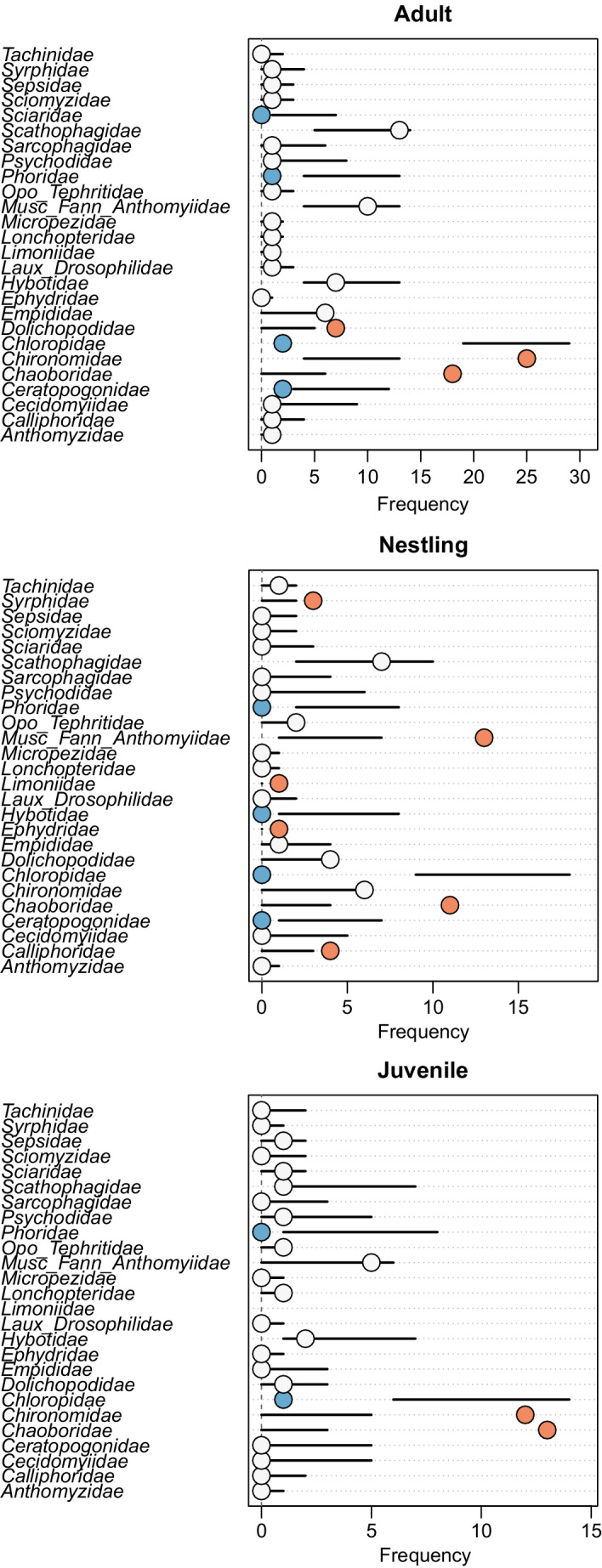
Dietary preferences of adult, nestling and juvenile reed warblers at Chew Valley Lake over the summer breeding season. Horizontal axes represent the number of fecal samples for a given reed warbler age class, whereas vertical axes list the Diptera prey families included in the model. Thick black lines represent predictions from the null model with 95% confidence limits, white circles represent Diptera prey families consumed in proportion to their availability, blue circles represent prey families eaten less frequently than expected and orange circles indicate prey families eaten more frequently than expected.

Predation rates on ~30% of Dipteran prey families were higher or lower than expected from the *econullnetr* null model when all age groups were combined, providing evidence for foraging preferences (Figure 5, but see Appendix S3 for full breakdown of effect sizes). Five families (15%) were consumed more than expected from their relative abundance by adults, six (24%) by nestlings, but in juveniles this was limited to two families (7%) (Figure 6). The midge family Chaoboridae was consumed more than expected by all age groups while chironomids were consumed more than expected by both adults and juveniles. Medium‐large bodied flies such as Calliphoridae, Dolichopodidae, Syrphidae, muscoid flies, and the limoniid crane flies were preferred by nestlings, and Dolichopodidae by both nestlings and adults. Reed warblers of all ages showed significantly fewer detections of small‐bodied flies such as Chloropidae, Ceratopogonidae, and Phoridae, than expected from the abundance of these families in the field.

## 4 DISCUSSION

### 4.1 Summary

Reed warbler diet varied between life stages, which should help to minimize competition while maximizing nutrition and fitness. Although dietary overlap was high, juveniles consumed a greater proportion of aquatic prey than either adults or nestlings, and nestlings consumed larger prey size classes overall. Moreover, each age group showed unique preferences for different Dipteran prey families, while the same suite of small‐bodied families was underexploited by all age groups. These dietary differences may allow competitive pressure to remain low, despite a high degree of prey sharing. We recommend the use of HTS for examining the diet of insectivorous species, and potentially other consumer groups, in detail over time and between age‐groups. Separately measuring the diets of animals at different developmental stages can aid the development of conservation measures to ensure essential prey availability is enhanced at sites, as well as inform distribution of a wide range of consumers.

### 4.2 Diet composition

While reed warblers consumed a very broad range of arthropods (192 species), we found that each age group occupied a measurably different dietary niche. Juvenile diet occupied an intermediate niche space between nestling and adult diet. Season and the interaction between age and season played a smaller role in structuring diet. Evidence for strong seasonality in dietary patterns was lacking, however diet composition did change subtly over the three stages of the breeding season, likely reflecting changes in invertebrate availability and richness.

Seasonal changes in diet have been demonstrated in metabarcoding studies on other insectivores (Clare et al., 2014; McClenaghan et al., 2019; Salinas‐Ramos et al., 2015; Tang et al., 2021), however, diets may also be influenced by interannual patterns of prey availability. For example, the occurrence of Coleoptera in the diet of big brown bats (*Eptesicus fuscus*) increased from early summer to late summer in one year, while the reverse pattern was observed in another year (Clare et al., 2014).

All age groups consumed a high diversity of prey, suggesting that a broad and generalist diet is common to all age stages. As expected, invertebrates from wetland habitats were a major food resource. Many insectivores are dependent on species of aerial insects that have an aquatic life stage (Baxter et al., 2005; Stanton et al., 2017; Trevelline et al., 2016), and the significance of aquatic prey in sustaining many insectivorous populations is well established in the literature (Bartels et al., 2012; Michelson et al., 2018; Newton, 1998).

Nonetheless, terrestrial groups formed roughly half of the diet of reed warblers at Chew Valley Lake. Reed warblers are strongly associated with wetlands, however, they also forage in a variety of terrestrial habitats, where there are opportunities to feed on terrestrial arthropods (Grim & Honza, 1996). This may be important, particularly when aquatic or other profitable preys are less available (Turner, 1982). For example, some insectivores show preferences for aquatic aerial insects but switch to terrestrial prey when the latter are higher in abundance (Michelson et al., 2018; Turner, 1982). In this study, the consumption of specific food resources showing temporary abundance is implied by the variation in diet across individuals and nests. This may be a result of switching between different taxa of temporarily abundant prey, between peaks of aerial insect emergence from the wetland environment.

### 4.3 Breeding currency and nestling prey resources

Differences between adult and nestling diet have been described in other songbirds. In a study by Durst et al. (2008), nestling southwestern willow flycatcher (*Empidonax traillii extimus*) diets had a higher proportion of Diptera compared to adult diets. In our study, nestling diet was species‐rich in large, soft‐bodied prey groups, including both Lepidoptera and Hemiptera, and detections of large‐bodied prey such as Odonata and Gastropoda were most frequent in nestling samples.

Lepidoptera was significantly more frequent in the diets of nestlings, aligning with the expectations of the breeding currency hypothesis. High prevalence of larval Lepidoptera is a common characteristic of nestling diet (Jedlicka et al., 2017; Krupa, 2004; Maziarz & Wesołowski, 2010; Wesołowski et al., 2019; Wesołowski & Neubauer, 2017; Xiong & Lu, 2014), as they are a high‐quality, nutrient‐rich food group, preferentially selected by parents to enhance offspring growth and increase breeding productivity (Greenberg, 1995; Skipper & Kim, 2013; Yard et al., 2004).

Preferential consumption of spiders is also often reported in nestling diet studies (Navalpotro et al., 2016; Pagani‐Núñez et al., 2011; Wesołowski et al., 2019). Spiders contain high concentrations of taurine, an amino acid which is essential for central nervous system development (Arnold et al., 2007; Ramsay & Houston, 2003). In this study, philodromid crab spiders were present in reed warbler nestlings' diet at a relatively high frequency, suggesting that this family may be important at this early stage of development.

### 4.4 Prey size is of key importance to nestling diet

Metabarcoding was unable to provide information about the life stage of the prey item consumed. Since intraspecific differences in size can occur in some taxa, our measures of prey size were subject to a degree of error. The only way to truly measure size differences is to measure prey items in fecal samples. Nonetheless, we believe our approach was sufficient for providing an approximate estimate of average size per taxon, allowing a simple comparison between age groups.

Although it might be expected that young birds would be fed manageable, smaller items, here nestlings consumed the largest size classes. In accordance with our findings, Grim and Honza (1996) found that the average body length of prey fed to reed warbler nestlings was 8 mm, close to our average of ~7 mm, and up to 21 mm. The larger prey items in our study generally represented softer bodied, high calorie taxa which are considered valuable nestling food (Skipper & Kim, 2013). Acrocephalids have been observed feeding their nestlings larger than average sized prey in bundles (Leisler et al., 2002) with those inhabiting more productive habitats able to capture larger prey and provision their young in fewer flights to the nest (Sejberg et al., 2000).

The nutritional requirements of a brood changes significantly from hatching to fledging (Jedlicka et al., 2017; Orlowski et al., 2015; Wesołowski et al., 2019; Wesołowski & Neubauer, 2017). All fecal samples from nestlings in this study were collected from individuals that were 7–10 days old, and by this age they can handle both larger and more chitinous prey than younger nestlings. Krupa (2004) showed that the daily number of feeds, number of prey items received, and the biomass of food increased over time as willow warbler (*Phylloscopus trochilus*) nestlings developed. Likewise, reed warbler parents may be under selective pressure to bring ever‐larger prey to their nestlings.

### 4.5 Juvenile diet and links to habitat associations

Juvenile birds consumed a greater proportion of prey with an aquatic life stage compared to either nestlings or adults, irrespective season. To our knowledge this difference has not been described in reed warblers before and while it is possible that this difference may be site‐ or year‐dependent, it may suggest foraging habitat separation. Earlier studies suggested that juvenile acrocephalids forage in a wider variety of habitats than adults (Barlein, 1981; Leisler & Shulze‐Hagen, 2011; Preiszner & Csorgo, 2008). If juveniles are using sub‐optimal foraging habitats, they may rely more on super abundances of emerging semi‐aquatic aerial insects that are provisioned by wetted zones. Alternatively, juveniles might show lower dietary plasticity than adults and be less able to adapt to the more seasonal prey resources found in terrestrial habitats. Monitoring individual birds in the field would allow the tracking of juvenile bird movements and confirm patterns in habitat separation that may lead to the observed dietary differences.

Foraging naivety and exploratory feeding behavior is expected from newly fledged birds (Marchetti & Price, 1989), which may explain why juveniles consumed a smaller suite of prey species than adult birds. Some prey groups may have proven difficult to capture, particularly those that fly at high speeds or are very large, such as the damselfly *Enallagma cyathigerum* which was consumed by adults and nestlings but not juveniles, despite being present throughout the summer months.

### 4.6 Dietary overlap among age classes and the effect of seasonality

Dietary overlap among the age classes was significantly greater than expected by chance. Although diet differed between age groups, it included several shared prey items, such as *C. flavicans*, *S. stercoraria*, and various species of chironomids, found at high abundances in the field. The presence of these consistently occurring prey in the diet likely constitutes a stable, plentiful food resource in all seasons (Clare et al., 2014). Foraging on locally abundant prey should serve to reduce the likelihood of intraspecific competition occurring (Rosenberg et al., 1982). The high degree of dietary similarity observed in the middle of the breeding season in this study is therefore unsurprising, since emergence of insects, notably Diptera, rises when summer temperatures are at their peak, enabling more widespread sharing of the most abundant prey groups (Davies, 2020). Trevelline et al. (2018) suggested a similar mechanism for the high levels of dietary overlap observed between nestlings of different riverine species coinciding with “super abundances” of emerging aquatic prey in wetlands.

In the early breeding season, overlap between nestlings and adults was low, though not significantly lower than the null model. Collection of more samples in the early season may allow us to clarify whether this reflects partitioning of prey or not. For example, reedbed primary productivity is limited by lower temperatures earlier in the year, and many invertebrate groups are not yet emerging in large numbers (Halupka et al., 2008; McKee & Richards, 1996). When there is a smaller suite of prey items to choose from, adults might be expected to maximize their reproductive output by capturing the most nutritious prey for their offspring, and forage themselves on less optimal prey as a trade‐off.

### 4.7 Dietary selectivity in reed warblers

Pearson et al. (2018) found evidence that consumers disproportionately consumed the most abundant species, supporting the theory that the key determinants of prey choice are encounter rate, capture success, handling efficiency, and nutritional quality (Symondson, 2002). Our study provides some evidence of several common Dipteran families being consumed at a higher rate than expected.

Adults and (notably) juveniles showed fewer preferences for different Dipteran families than nestlings, suggesting that the foraging strategy of both might involve opportunistically consuming encountered invertebrates. In contrast, nestling diet is likely to be a result of parents selecting the most nutritionally valuable prey available, and at times these may represent less abundant prey items.

In a recent study, McClenaghan et al. (2019) demonstrated with metabarcoding that barn swallows (*Hirundo rustica*) select larger prey items from a small subset of Dipteran families to feed their nestlings. Similarly, here, several large‐bodied fly families, such as hoverflies, muscoid flies, and crane flies were disproportionately selected for reed warbler nestlings in relation to their abundance on sticky traps.

Both here and in other studies, birds supplemented their diet with high value prey that are limiting in the environment, or only temporarily available, over more abundant, and readily available groups, provided that the latter are smaller‐bodied and less nutritious (Bibby & Thomas, 1985; Durst et al., 2008; Rytkönen et al., 2019; Yard et al., 2004). In contrast, the universal consumption of the often‐abundant aquatic midges Chironomidae and Chaoboridae, beyond what would be expected from their measured availability, reiterates the importance of large numbers of aquatic prey for sustaining insectivores.

### 4.8 Climate change implications

Reed warblers have advanced both their arrival on the breeding grounds and their onset of reproduction as a direct result of climate change. This has resulted in an extended breeding season associated with positive impacts on productivity and fitness (Eglington et al., 2015; Halupka et al., 2008; Schaefer et al., 2006). Although the long‐term consequences of such warming for insectivores is still little understood, increasingly warm springs in reed beds will change patterns of invertebrate emergence and their subsequent availability to reed warblers (Baillie et al., 2013; Kampichler & van der Jeugd, 2011; Vafidis, 2014; Vafidis et al., 2016). Given the high prevalence of wetland‐associated prey in warbler diets, the widespread adoption of conservation and/or restoration of wetland habitats is essential to ensure that a plentiful supply of emerging insects is available to insectivore populations.

### 4.9 Metabarcoding limitations

Our primer set is longer than is typically recommended for metabarcoding (Clare, 2014), and it is possible that very degraded prey DNA may have been under‐represented in our study. In practice, however, the primer set had very wide utility, amplifying a wide diversity of invertebrates, including mollusks, arachnids, and insects. The primers do not amplify warbler DNA, so we did not lose any reads to the host's own DNA, which was a great advantage. To minimize losses of zOTUs present in mixed fecal samples, we used a v3 chip to ensure a deep level of sequencing and a high number of retained reads. While we recommend this approach for future applications of this primer pair, it is important to note that no primer pair alone can provide a completely unbiased and comprehensive account of an animal's diet (Pompanon et al., 2012).

Modifications to the extraction protocol were beneficial in reducing substantial amounts of PCR inhibiting uric acid from nestling fecal sacs. Complete removal could not always be achieved, however, reflected in the lower amplification success rate for nestling samples. This finding may be useful for future dietary studies and demonstrates the need for testing specific protocols designed to reduce PCR failure rate from inhibitors.

## 5 CONCLUSION

DNA metabarcoding is a highly effective tool for dietary analysis, generating fine‐scale taxonomic information (Alonso et al., 2014; Soininen et al., 2009). By using this technique, we have contributed species‐level detail to reed warbler diet, which previous studies lacked. Our study also sheds light on the possible mechanisms for dietary selectivity (of insect prey) by migratory songbirds at their breeding sites. Since reed warblers are a typical example of a trans‐Saharan migratory species, our methodology and subsequent findings may be useful for inferring the feeding biology of other passerines with similar life histories, particularly with respect to changes in prey availability as a result of on‐going and future environmental change. Reed warblers consume a very wide breadth of prey, and a long‐term project spanning multiple years, with a larger sample size for each age group, would be needed to accurately measure dietary variation over longer temporal scales. Nonetheless, we believe this study expands our knowledge of how consumers of different age stages interact with their prey both in dynamic environments and in the presence of congeners.

## AUTHOR CONTRIBUTIONS

**Sarah R. Davies:** Conceptualization (equal); data curation (lead); formal analysis (lead); investigation (lead); methodology (equal); project administration (lead); visualization (lead); writing – original draft (lead); writing – review and editing (equal). **Ian P. Vaughan:** Conceptualization (equal); formal analysis (supporting); funding acquisition (equal); investigation (supporting); methodology (equal); supervision (equal); validation (equal); writing – review and editing (equal). **Robert J. Thomas:** Conceptualization (equal); formal analysis (supporting); funding acquisition (equal); investigation (supporting); methodology (equal); supervision (equal); validation (supporting); writing – review and editing (supporting). **Lorna E. Drake:** Formal analysis (supporting); methodology (supporting); resources (equal); validation (supporting); writing – review and editing (supporting). **Angela Marchbank:** Data curation (supporting); methodology (supporting); resources (equal); supervision (supporting); validation (supporting); writing – review and editing (supporting). **William O. C. Symondson:** Conceptualization (equal); formal analysis (supporting); funding acquisition (lead); investigation (equal); methodology (equal); resources (equal); supervision (lead); validation (equal); writing – review and editing (equal).

## CONFLICT OF INTEREST

The authors declare they have no conflict of interest.

## BENEFIT‐SHARING STATEMENT

Benefits from this research accrue from the sharing of our data and results on public databases as described above. All samples were collected, and data generated in the author's country of birth. The thesis from which this publication is based is freely available on Cardiff University's repository: Online Research at Cardiff (ORCA) and has been shared with collaborators. The contributions of collaborating volunteers (bird ringers) from the BTO who assisted with collecting samples are described in the Section 2 and Acknowledgments.

## Supporting information


Appendix S1

Appendix S2

Appendix S3

Appendix S4

## Data Availability

Sample metadata (including georeferences and date/month/year of sampling event) and R scripts for statistics are freely available for download on Zenodo (metadata at https://doi.org/10.5281/zenodo.5803128 and R scripts at https://doi.org/10.5281/zenodo.6839108). Raw DNA sequence files are available at Dryad: https://doi.org/10.5061/dryad.wpzgmsbqm. [Link], [Link] are available online with the manuscript. Other data are available on request upon contacting the corresponding author.
