# Supplementary material for: Seasonal and ontological variation in diet and age‐related differences in prey choice, by an insectivorous songbird
Source: Ecol Evol. 2022 Aug 11;12(8):e9180. doi: 10.1002/ece3.9180 (PMC9366593; doi:10.1002/ece3.9180)

# **Supplementary Materials 1-7**

## **S1. Modifications to the QIAGEN QIAmp® DNA Stool Mini Kit protocol and steps taken to reduce the risk of contamination**

DNA extraction from warbler faecal material was carried out following the standard protocol, including all recommended steps with modifications by Zeale et al. 2011, Nicholls 2017 (pers comm) and Shutt et al. 2020. The following modifications were used;

1. Uric acid was removed from each stool sample by scraping the sides of the faecal pellet. Either the whole pellet, or up to 220mg of the pellet (if sample is large) was used in the extraction. Samples were removed from ethanol and left to air dry before being added to a new microcentrifuge tube.
2. 500μl of InhibitEx Buffer was added to each stool sample, then the sample was mixed manually using a pestle for 20 seconds before adding a further 500μl of InhibitExBuffer. The samples were then homogenized by vortexing for 3-4 minutes and incubating for 10-15 minutes in a water bath at 70°C. Samples were vortexed again for 2 minutes to resuspend.
3. 20μl of proteinase K, 400μl of supernatant and 400μl Buffer AL was added to a new tube in step 5 and samples were incubated at 70°C for 20 minutes before adding 400μl molecular grade (96-100%) ethanol.
4. 80μl of Buffer ATE (or AE) was added to each spin column membrane, or 60μl for samples with small amounts of faecal material. Samples were incubated at room temperature for 5-10 minutes and then centrifuged at full speed for 1 min to elute DNA. To increase DNA yields we repeated this step by transferring the eluate back into the original spin column within a new tube and centrifuged at full speed for another 1 min before eluting DNA.

During extraction and PCR steps, several steps were taken to reduce the risk of contamination from outside sources. All faecal extractions were carried out under an air flow hood that was cleaned thoroughly between uses with bleach and ethanol. DNA extractions from invertebrate tissue were carried out in a separate location to PCR set up areas to prevent cross contamination. Eppendorf tubes and pestles were autoclaved before use, and these and all pipettes, racks and other tools used in the extraction were cleaned with bleach and placed under ultraviolet light for 20 minutes prior to extraction. Tools for removing uric acid, cutting and transferring samples, were sterilised between samples by washing in bleach, water and ethanol and flaming with a Bunsen burner torch. A new, clean pestle was used for breaking up each sample.

All PCRs were carried out under conditions to reduce contamination risk. Work surfaces were thoroughly cleaned, and equipment/materials to be used in the setup were autoclaved and/or cleaned with bleach and placed under ultraviolet light for 20 minutes prior to PCR set up. Separate pipettes were used for adding reagents in PCR setup and for adding DNA, and all non-DNA reagents were removed from the area before DNA aliquots were added. Setup for MID-tag PCR took place under an air flow hood to further reduce the risk of contamination. All plates contained extraction negatives (spread across the plate) and a column of PCR negatives (nuclease free water) to test for the presence of contamination in each row. Any rows containing a PCR negative sample that tested positive for DNA were not taken forward and reactions were repeated to ensure that samples were free from contamination.

## **S2. List of forward and reverse MID-tag oligos used for metabarcoding**

| FF02 | ACGCTCGACA |  |  | RR01 | ACTAGCAGTA |
| --- | --- | --- | --- | --- | --- |
| FF03 | AGACGCACTC |  |  | RR04 | TGTGAGTAGT |
| FF04 | AGCACTGTAG |  |  | RR05 | TGACGTATGT |
| FF05 | ATCAGACACG |  |  | RR07 | TCTAGCGACT |
| FF06 | ATATCGCGAG |  |  | RR08 | TCGCACTAGT |
| FF07 | CGTGTCTCTA |  |  | RR09 | TCGATCACGT |
| FF08 | CTCGCGTGTC |  |  | RR10 | TAGTGTAGAT |
| FF10 | TCTCTATGCG |  |  | RR11 | TACGCTGTCT |
| FF11 | TGATACGTCT |  |  | RR12 | TACAGATCGT |
| FF13 | CATAGTAGTG |  |  | RR13 | TACACGTGAT |
| FF15 | ATACGACGTA |  |  | RR14 | TACACACACT |
| FF16 | TCACGTACTA |  |  | RR15 | CGACGTGACT |
| FF17 | CGTCTAGTAC |  |  | RR16 | CAGTAGACGT |
| FF18 | TCTACGTAGC |  |  | RR17 | CACGCTACGT |
| FF19 | TGTACTACTC |  |  | RR18 | ATAGAGTACT |
| FF20 | ACGACTACAG |  |  |  |  |
| FF21 | CGTAGACTAG |  |  |  |  |
| FF22 | TACGAGTATG |  |  |  |  |
| FF23 | TACTCTCGTG |  |  |  |  |
| FF24 | TAGAGACGAG |  |  |  |  |
| FF25 | TCGTCGCTCG |  |  |  |  |
| FF26 | ACATACGCGT |  |  |  |  |
| FF27 | ACGCGAGTAT |  |  |  |  |
| FF30 | AGACTATACT |  |  |  |  |
| FF31 | AGCGTCGTCT |  |  |  |  |

## **S3. Mock community composition**

Table S.3. Mock community mixes included in the HTS run. Communities were composed of DNA samples from tissue extracts from the invertebrates listed below. All invertebrate DNA sample concentrations were standardised at 0.1 ng/µL^-1^ by diluting the DNA in DNase-free water. Values indicate the volume in microlitres (μL) of each DNA sample added to the mock community mix. The bottom row indicates the final total volume of each mix. Green background indicates species from each mix that were subsequently detected with high-throughput sequencing (after all bioinformatics and data clean-up steps), red indicates species that were not detected.

1. Composition of mock community mixes 1-5: DNA extracts from collected invertebrates.

|  |  | Volume added to mix (μL) | | | | |
| --- | --- | --- | --- | --- | --- | --- |
| Taxon Description | ID | Mix1 | Mix2 | Mix3 | Mix4 | Mix5 |
| Springtail | Unknown sp. | 1 | 1 | 3 | 1 | 2 |
| Assassin bug | *Anthocoris nemorum* | 1 | 2 | 3 | 1 | 1 |
| spider | *Erigone dentipalpis* | 1 | 1 | 3 | 1 | 2 |
| wasp | *Trichopria sp.* | 1 | 2 | 3 | 1 | 1 |
| spider | Unknown sp. | 3 | 1 | 1 | 1 | 2 |
| aphid | *Utamphorophora sp.* | 3 | 2 | 1 | 1 | 1 |
| parasitoid | *Promethes sulcator* | 3 | 1 | 1 | 1 | 2 |
| springtail | *Orchesella villosa* | 1 | 2 | 3 | 1 | 1 |
|  |  | 14 | 12 | 18 | 8 | 12 |

## **S4. Outline of bioinformatics pipeline and data clean up**

Paired-end Illumina reads were trimmed, aligned and checked for quality using FastP v.0.20.0 (Chen et al. 2018) using a minimum read length of 200bp and a minimum base quality threshold score of 33. Mothur (Schloss et al. 2009) assigned MID-tag labelled reads to their respective sample ids and removed the primer sequences. Sequences were checked in both directions and a minimum of one mismatch was permitted. A demultiplexing step sorted the resulting reads into one file per sample id. Each read was given a header of the respective sample ID before being concatenated into a single file. The Unoise3 command (Edgar and Flyvbjerg 2015, Edgar 2016) in Usearch v.11 (Edgar 2010) was then performed using a clustering threshold of 100% to [i] remove chimeras and noise, [ii] cluster the reads to generate denoised zero-radius OTUs (zOTUs) and [iii] create a read abundance matrix for samples and zOTUs.

For a given zOTU to be retained in a sample, its read count needed to exceed a threshold of 0.2% of the maximum read count for all zOTU sequences from that sample. This threshold was determined by finding the % values at which artefacts present in the known mock community samples were removed while still retaining all known species in the mock community (Drake et al. 2021, Cuff et al. 2021). To reduce the incidence of tag jumping from zOTUs with very high read counts across samples, a zOTU present in a sample needed to comprise a read count of more than 0.5% of the maximum read count for all incidences of that taxa across all samples in order to be retained. Steps carried out were [i] extract the maximum read values found in PCR and DNA extraction negatives and the MID-tag combinations that were not used in any reaction in the study (termed NAs), [ii] remove zOTUs from samples where the read number was lower than the maximum value found in the negatives and NAs and/or where the 0.2% and 0.5% thresholds specified above were not met; and [iii] collapse and aggregate the matrix so that all prey species detections for each zOTU are represented by a single taxonomic entry.

## **Shell and perl scripts for metabarcoding data used in the bioinformatics pipeline**

The following scripts were written, modified and recommended by Drake et al. 2021. The entire pipeline was repeated for each indexing pool (pool 1 shown).

*Script 1 – Trimming and aligning paired reads to generate complete amplicon sequence*

## we will do FastQC quality check, merge the paired end reads and trim the sequences in one go using FastP to get the complete amplicon sequence

/mnt/data/GROUP-sabwocs/c1618864/fastp -i SD-CO1-1-290519_S1_L001_R1_001.fastq -I SD-CO1-1-290519_S1_L001_R2_001.fastq -l 200 -m --discard_unmerged -o pool1merged.fastq

## next convert the fastq file to fasta format

module load fastx_toolkit/0.0.14

fastq_to_fasta -i pool1merged.fastq -Q 33 -o pool1merged.fa

*Script 2 – Allocate MID-tag combinations to their respective samples and remove primer sequences*

## we will identify the sequences that match the oligos used, allowing for 1 mismatch. oligos = text file where the first column reads #‘primer’, the second and third columns are the forward and #reverse primer and MID-tag combinations for a particular #sample, and the fourth column is the sample ID annotated with #an additional ‘a’ or ‘b’. ‘a’ is used when the forward primer #is in column 2 and the reverse is in column 3. ‘b’ is used #when this order is reversed. This means that the total number #of rows should be twice the number of samples.

#Run Mothur

module load mothur/1.39.5

mothur "#trim.seqs(fasta=merged.fa,oligos=oligo1.txt,checkorient=t,pdiffs=1)"

#split .groups file into A and B

grep 'a$' merged.groups > mergedA.groups

grep 'b$' merged.groups > mergedB.groups

#remove 'a' and 'b' labels

sed -i 's/a//g' mergedA.groups

sed -i 's/b//g' mergedB.groups

*Script 3 – Demultiplexing*

*Part 1. Perl script*

#!/usr/bin/perl

# fastalist1.txt is a text file that is identical to the fourth column of the oligos file described in Script 3

unless ($#ARGV == 0)

{

print "Usage: 3_Demultiplex.pl fastalist1.txt";

die;

}

open (INLIST, "<$ARGV[0]") || die;

# replace 'XXX' with your username, and if you want to put the output into another directory you can add that to the 'outdir' path here

$indir = "/mnt/data/GROUP-sabwocs/c1618864/pool1/deplexed";

$outdir = "/mnt/data/GROUP-sabwocs/c1618864 /pool1/deplexed";

# Loops through the list for your samples ('SampleList') and performs the commands for each one

while (<INLIST>) {

$lib = $_;

chomp($lib);

# A shortcut to read or write a file for each of your samples, each file having the same extension

$readidsa = $lib . "_a_ids.txt";

$readidsb = $lib . "_b_ids.txt";

$readidsab = $lib . "_ab_ids.txt";

$fa1 = $lib . ".fa";

$fa2 = $lib . ".fasta";

# split fasta read IDs into files grouped by sample ID. Replace 'XX' with the name of you '.groups' file (output from mothur)

system("grep -w $lib $indir/mergedA.groups | awk '{print \$1}' > $outdir/$readidsa");

system("grep -w $lib $indir/mergedB.groups | awk '{print \$1}' > $outdir/$readidsb");

# combine the list of sequence names for 'a' and 'b' matches

system("cat $outdir/$readidsa $outdir/$readidsb >> $outdir/$readidsab");

# split the trimmed fasta file into reads specific to each sample. Replace 'XX' with the name of your trimmed fasta file (output from mothur)

my $command1 = 'perl -ne'."'".'if(/^>(\S+)/){$c=$i{$1}}$c?print:chomp;$i{$_}=1 if'." @ARGV'"." $outdir/$readidsab $indir/merged.trim.fasta > $outdir/$fa1";

system ($command1);

system("awk '{print \$1}' $indir/$fa1 > $indir/$fa2");

}

exit;

*Part 2. Shell script*

perl 3_Demultiplex.pl fastalist1.txt

*Script 4. Editing header information for each sample*

*Part 1. Perl script.*

#!/usr/bin/perl

unless ($#ARGV == 0)

{

print "Usage: 4_Edit_Headers.pl fastalist1.txt";

die;

}

open (INLIST, "<$ARGV[0]") || die;

$indir = "/mnt/data/GROUP-sabwocs/c1618864/pool1/deplexed/fastafiles";

$outdir = "/mnt/data/GROUP-sabwocs/c1618864/pool1/deplexed/fastafiles";

while (<INLIST>) {

$lib = $_;

chomp($lib);

$fa1 = $lib . ".fasta";

$fa2 = $lib . "_edit.fasta";

system( qq(sed "s/^>/>$lib;/g" "$indir/$fa1" > "$indir/$fa2"));

}

exit;

*Part 2. Shell script*

perl 4_Edit_Headers.pl fastalist1.txt

*Script 5 - Concatenate all sequences into one file*

cat *edit.fasta > Allmerged.fasta

*Script 6 – USEARCH*

# removes identical replicates from the fasta input, output for next step =SampleName_rc_uniques.fasta

/mnt/data/GROUP-sabwocs/c1618864/pool1 -fastx_uniques pool1.fasta -fastaout Unique.fasta -sizeout -strand both -relabel Uniq -threads 4

# sort by size

/mnt/data/GROUP-sabwocs/c1618864/pool1 -sortbysize Unique.fasta -fastaout Sorted.fasta

# Cluster OTUs

/mnt/data/GROUP-sabwocs/c1618864/pool1 -cluster_otus Sorted.fasta -otus OTU.fasta -relabel Otu

# denoise and cluster using unoise3 to make zOTUs

/mnt/data/GROUP-sabwocs/c1618864/pool1 -unoise3 Sorted.fasta -zotus zOTU.fasta

# make matrix of zOTU's and the number of sequences per zOTU (size)

/mnt/data/GROUP-sabwocs/c1618864/pool1 -otutab pool1.fasta -zotus zOTU.fasta -otutabout zOTUtable_COI.txt -strand both -threads 4

# make matrix of OTU's and the number of sequences per OTU (size)

/mnt/data/GROUP-sabwocs/c1618864/ pool1 -otutab pool1.fasta -otus OTU.fasta -otutabout OTUtable_COI.txt -strand both -threads 4

*Script 7. BLAST*

# blast the clusters from usearch

module load blast/2.7.1

export BLASTDB=/mnt/data/GROUP-sabwocs/c1618864/pool1/BLAST-DB

blastn -query zOTU.fasta -db nt -num_threads 4 -evalue 0.00001 -perc_identity 97 -outfmt 6 -out zOTU.blastOutput.txt

blastn -query OTU.fasta -db nt -num_threads 4 -evalue 0.00001 -perc_identity 97 -outfmt 6 -out OTU_blastOutput.txt

*Script 8. Filter the BLAST results*

# only keep results with over 95% identity and remove and sequences with less than 100bp in length

awk ‘$3 >= 95’ OTU_blastOutput.txt | awk ‘$4 >= 100’ > OTU_blast_filtered.txt.

awk ‘$3 >= 95’ zOTU_blastOutput.txt | awk ‘$4 >= 100’ > zOTU_blast_filtered.txt.

*Script 9. Add taxon information to diet zOTU matrix (R-script)*

#Add in taxon information to your zOTU and OTU tables: Open R and run the following code on your blast output to get only the top hit for each motu based on bitScore (combination of e-value and percentage identity):

>blast <- read.table("zOTU_blastOutput.txt")

>summary(blast)

>library(dplyr)

>blast_filter <- blast %>%

group_by(V1) %>%

filter(V12 == max(V12))

>write.table(blast_filter, "pool1_zOTU_TopHit_blastOutput.txt")

#Next use the program MEGAN to assign ids to each zOTU from the BLAST top hit output.

#Use VLOOKUP in Excel to add taxon ids to each zOTU in the diet matrix.

#Calculate maximum contamination/tag jumping from NAs and negative controls and remove the same value from all reads along each row. Convert negative values to 0.

#Remove all reads with a read count of less than 10.

#Remove zOTUs that are at their highest read count in positive controls and mock communities from the remaining diet matrix.

#Remove non-dietary data

#Convert matrix to csv file for aggregating in R.

*Script 10. Aggregate zOTUs in diet matrix based on taxon id (R-script)*

>pool1_to_Agg <- read.csv(“zOTUtable_COI.csv”, header = T)

>Agg <- aggregate(.~Taxon, data=pool1_to_Agg, sum)

>write.table(Agg, “pool1_Aggregated.csv”)

## **S5. Taxa removed from the metabarcoding datasets**

Table S.5. Species removed from the dietary datasets during the data clean-up steps subsequent to sequence identification with BLAST and BOLD. The taxon species name, common name are given along with the respective reasons for removal and GenBank accession code.

| Taxon | Common Name | Reason for removal | Accession Code |
| --- | --- | --- | --- |
| *Arthrodermataceae sp.* | Fungi | Not dietary | MG592681.1 |
| *Cladosporium sp.* | Fungi/mould | Not dietary | FJ590524.1 |
| *Cercospora sojina* | Fungal plant pathogen | Not dietary | KC888822.1 |
| *Penicillium chrysogenum* | Pennicilin fungus | Not target taxa | AM920464.1 |
| *Penicillium cinnamopurpureum* | Penicillin fungus | Not target taxa | FJ004561.1 |
| *Penicillium citrinum* | Penicillin fungus | Not target taxa | EF180187.1 |
| *Penicillium janthinellum* | Penicillin fungus | Not target taxa | FJ004537.1 |
| *Penicillium nordicum* | Penicillin fungus | Not target taxa | KR952336.1 |
| *Penicillium polonicum* | Penicillin fungus | Not target taxa | KU530219.1 |
| *Penicillium sp.* | Penicillin fungus | Not target taxa | FJ004537.1, KU530219.1 |
| *Saprolegnia ferax* | Water mould | Not target taxa | KM361513.1 |
| *Bremia sonchicola* | Brown algae | Not dietary | MF687314.1 |
| *Pernospora aparines* | Oomycete | Not target taxa | HM033187.1 |
| *Pernospora romanica* | Oomycete | Not target taxa | KJ654123.1 |
| *Achlya hypogyna* | Oomycete | Parasite, not target taxa | KF226724.1 |
| *Pythium sp.* | Oomycete | Not target taxa | JN660054.1 |
| *Oomycetes sp.* | Oomycete | Parasite, not target taxa | HQ708212.1 |
| *Leptolegnia sp.* | Oomycete | Not target taxa | HQ708212.1 |
| *Proctophyllodes sylviae* | Feather mite | Not dietary | KU203163.1 |
| *Trouessartia trouessarti* | Feather mite | Not dietary | KP193817.1 |
| *Steinernema intermedium* | Nematode of invertebrates | Not dietary | JN808126.1 |
| *Arenicola marina* | Sandworm | Present only in positive controls | HQ691225.1 |
|  | Invertebrate environmental sample | Unclear identification | LC222761.1 |
| *Tetrastemma candidum* | Ribbon worm | Not dietary | AY791973.1 |
| *Eratigena duellica* | Giant house spider | Known lab contaminant - present in positive controls only | LT970989.1 |
| *Pterostichus melanarius* | Strawberry ground beetle | Known lab contaminant – present in positive controls only | DQ063219.1 |
| *Blattella germanica* | German cockroach | Known lab contaminant | EU854321.1 |
| *Pacifastacus leniusculus* | Signal crayfish | Present only in positive controls | KY947333.1 |
|  | Zooplankton environmental sample | Not dietary | KC732416.1 |
| *Acanthamoeba sp.* | Amoeba | Pathogen, not dietary | MG924682.1 |
| *Nitzschia palea* | Marine diatom | Not dietary | AP018512.1 |

## **S6. Invertebrate families that were combined in morphological identification of sticky trap samples and the subsequent *econullnetr* analysis**

| Families combined | Order | Morphological characteristics | Name code in *econullnetr* |
| --- | --- | --- | --- |
| Lauxaniidae,  Drosophilidae | Diptera | Both acalyptratae flies, similar coloration, superficial resemblance | Laux_Drosophilidae |
| Muscidae,  Fanniidae,  Anthomyiidae | Diptera | Calyptratae flies of the superfamily Muscoidea – similar wing venation and body plan | Musc_Fann_Anthomyiidae |
| Opomyzidae,  Tephritidae | Diptera | Both acalyptratae flies, similar wing markings, superficial resemblance | Opo_Tephritidae |

**S7. References for invertebrate body lengths**

Online sources, specialist websites and invertebrate keys were used to find estimates for the body length (in millimetres) of each prey species detected in our study (web resources by order: Araneae <https://araneae.nmbe.ch/>, Lepidoptera: ukmoths.org.uk/, Diptera: <http://diptera.info>, Coleoptera: [www.coleoptera.org.uk](http://www.coleoptera.org.uk), Hemiptera: [www.britishbugs.org.uk](http://www.britishbugs.org.uk), others: [www.bugguide.net](http://www.bugguide.net)). The average body length from all other species or genera in the family was used when species-specific information could not be obtained.

**S8. NMDS plots with the scores for the prey community (at the family level) plotted alongside the sample scores for the diets of Chew Valley reed warblers according to i) age and ii) season.**

**
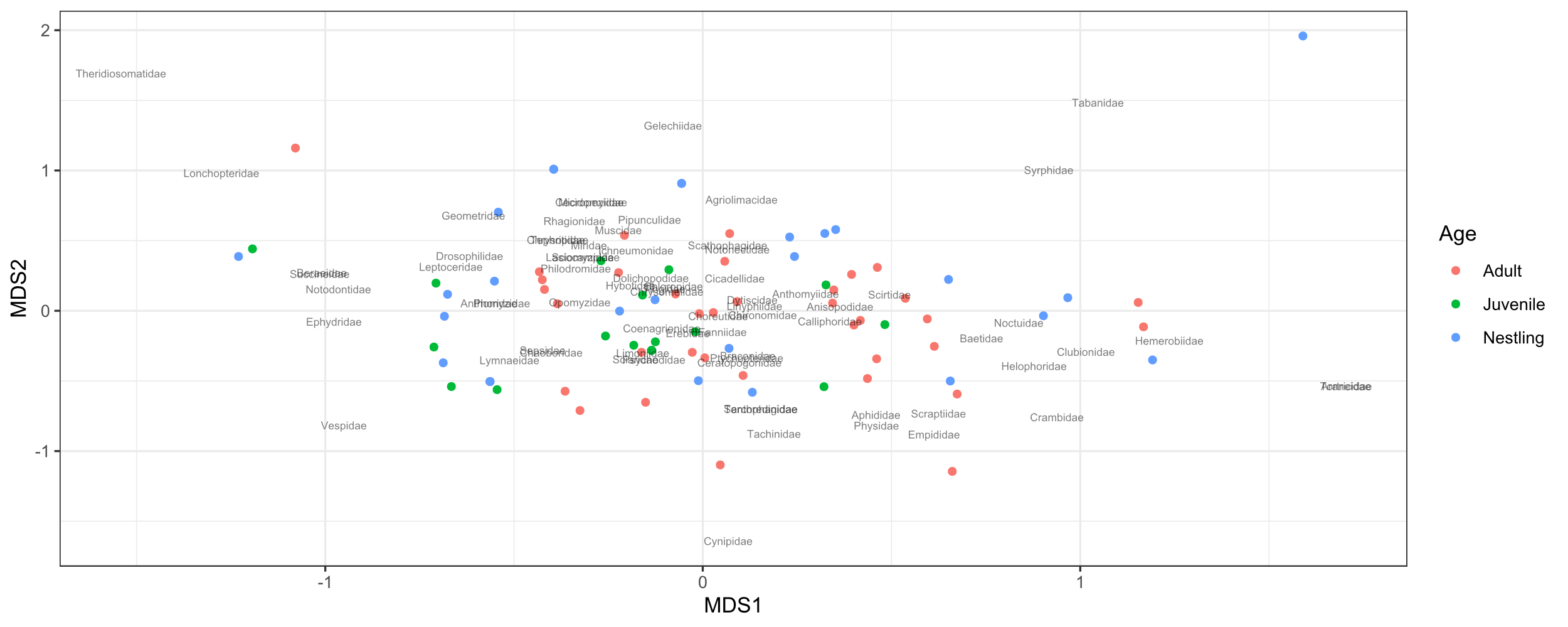
i)**

**ii)**


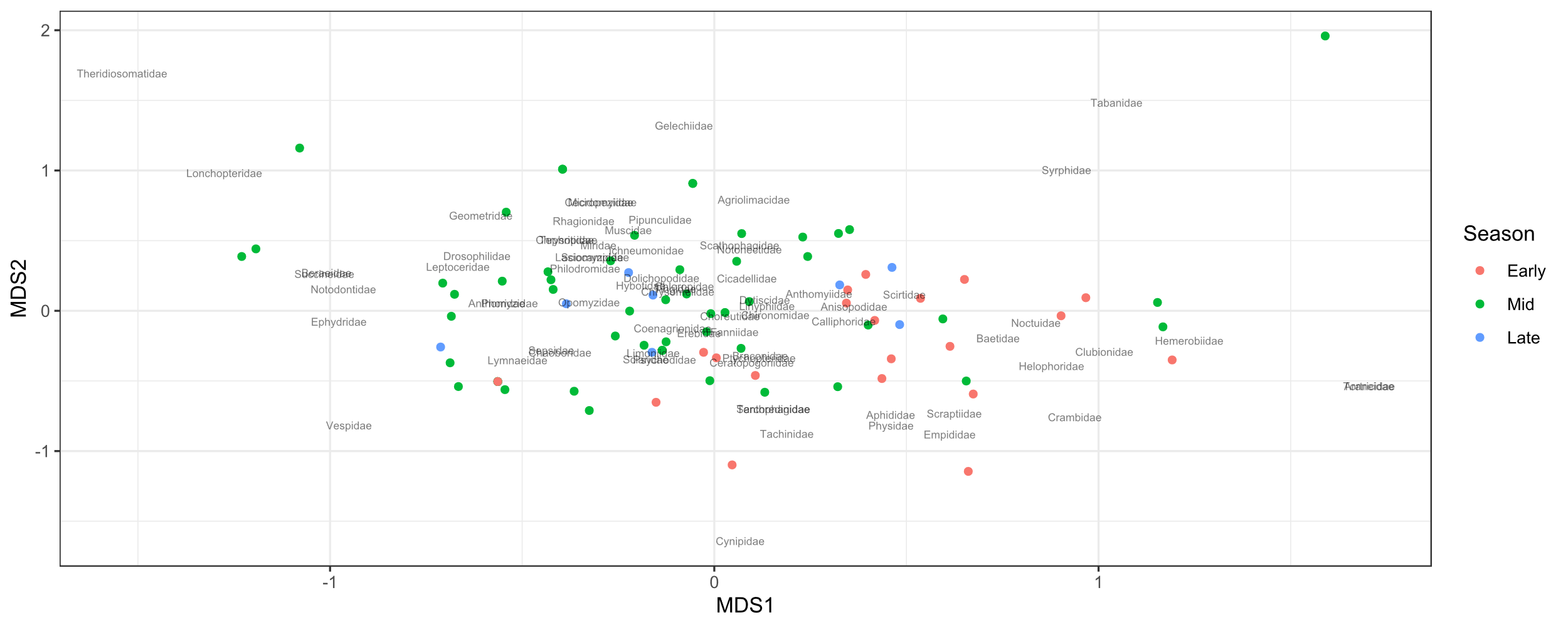

Supplement: Supplementary file 1 — Appendix S1 [file ECE3-12-e9180-s001.docx]
